# Lung Cancer Screening With Low-Dose CT: A Systematic Review

**DOI:** 10.7759/cureus.75515

**Published:** 2024-12-11

**Authors:** Pedro Pacheco, Vanda Melo, Cátia Martins, Helena Ribeiro

**Affiliations:** 1 Family Health Unit New Directions, Unidade Local de Saúde do Alto Ave, Vizela, PRT

**Keywords:** low-dose ct, lung cancer, mortality, screening, tomography

## Abstract

Lung cancer is highly prevalent worldwide and is the leading cause of cancer-related death in Portugal. There is increasing evidence that low-dose computed tomography (LDCT) screening reduces mortality; however, few countries have implemented screening strategies. This review aims to gather the best evidence to assess the relevance of implementing lung cancer screening. A search was conducted for clinical practice guidelines (CPGs), systematic reviews (SRs), and meta-analyses (MAs) published between January 1, 2010, and January 31, 2024, as well as randomized controlled trials (RCTs) published between January 1, 2019, and January 31, 2024, indexed in databases such as the National Guideline Clearinghouse, Cochrane Library, Guideline Finder, Canadian Medical Association, Evidence-Based Medicine Online, Database of Abstracts of Reviews of Effectiveness (DARE), and PubMed. The MeSH terms used were “lung cancer” and “screening”. To evaluate the level of evidence (LE) and strength of recommendation (SR) in the included MAs, the Strength of Recommendation Taxonomy (SORT) from the American Academy of Family Physicians was applied. A total of 460 articles were found, with two CPGs, six MAs, two SRs, and one RCT being selected. The CPGs recommend screening with LDCT for smokers with a smoking history of more than 20 pack-years, aged between 50 and 80 years. All MAs show statistically significant evidence of reduced mortality in screened patients, although without a reduction in all-cause mortality. However, there was some heterogeneity regarding the age of the target population and the screening follow-up period. Overdiagnosis rates varied between MAs. The SRs and RCT also demonstrated a reduction in lung cancer mortality, but not in all-cause mortality. LDCT lung cancer screening shows a reduction in disease-related mortality, suggesting that the implementation of organized screening for at-risk populations could have a significant positive impact. Some uncertainties remain regarding the best strategy for implementing organized screening programs.

## Introduction and background

Lung cancer is a highly prevalent disease worldwide, being the second most common cancer in men, following prostate cancer, and the second in women, following breast cancer, according to 2018 data, when 2,094,000 cases were diagnosed [1]. In Portugal, 5,415 new cases of lung cancer were diagnosed in 2020, with a total of 4,797 deaths from this disease, making lung cancer the third most incident cancer and the leading cause of cancer-related death in our country [2], as well as the sixth leading cause of death or disability from all causes [3]. Due to its high prevalence, lung cancer is also associated with significant costs for health systems. It is estimated that cancers of the respiratory tract (trachea, bronchi, and lung) account for the largest share of healthcare expenditures for oncological diseases, with 15.4% of costs, equivalent to approximately 3.9 trillion dollars globally [4].

Considering these numbers, it becomes important to discuss strategies to reduce the burden of this disease, specifically screening. Increasing scientific evidence has demonstrated the effectiveness of using low-dose computed tomography (LDCT) for early detection of lung cancer, as well as in reducing mortality. However, few countries have organized screening programs. In Europe, only Croatia, Poland, Italy, and Romania are formally committed to implementing screening strategies for at-risk individuals [5]. Tobacco is undoubtedly the most important risk factor for lung cancer, especially in smokers, but also in people exposed to passive smoking [6]. Therefore, the population exposed to a significant tobacco burden are the potential primary beneficiaries of screening strategies. However, several other factors have been identified, such as family history, previous lung disease (especially chronic obstructive pulmonary disease), occupational exposure to carcinogens (asbestos, silica, arsenic, beryllium, cadmium, etc.), pollution, estrogens, diet, and obesity [7,8]. Nevertheless, there are also some limitations highlighted in different studies regarding the use of LDCT for lung cancer screening. One limitation is the existence of studies that report a high rate of false positives, which may have implications for conducting more invasive and unnecessary subsequent diagnostic investigations [9]. Another potential limitation is that the criteria for selecting individuals for this screening are based on exposure rather than demographic data such as age and sex (as in other screenings currently implemented in Portugal), which may imply self-selection that is often biased [10].

Thus, this review aims to evaluate the scientific evidence to determine whether lung cancer screening with LDCT, applied to the adult smoking population, reduces mortality from lung cancer.

## Review

Methods

A search was conducted for clinical guidance standards (NOCs), systematic reviews (SRs), and meta-analyses (MAs) published between January 1, 2010, and January 31, 2024, randomized controlled trials (RCTs) published from January 1, 2019, to January 31, 2024, and indexed in the National Guideline Clearinghouse databases, Cochrane Library, Guidelines Finder, Canadian Medical Association, Evidence-Based Medicine Online, Database of Abstracts of Reviews of Effectiveness (DARE), and PubMed. The following MeSH terms were used: “lung cancer” and “screening”.

Inclusion criteria for articles were based on the target population consisting of adult smokers; articles written in Portuguese, English, or Spanish; and lung cancer screening by low-dose pulmonary CT compared to screening by chest radiography or no screening. The primary outcome was the reduction in mortality from lung cancer, and secondary outcomes were the reduction in mortality from all causes and the rate of overdiagnosis.

Exclusion criteria included non-RCTs, clinical trials included in the selected SRs or MAs, duplicate articles, and articles unrelated to the review's objective.

In evaluating the level of evidence (LE) and strength of recommendation (SR) of the included MAs, the Strength of Recommendation Taxonomy (SORT) scale from the American Academy of Family Physicians was applied. To assess the SR of the included NOC, the scale adopted by the authors of the original documents was used.

Results

**Figure 1:**
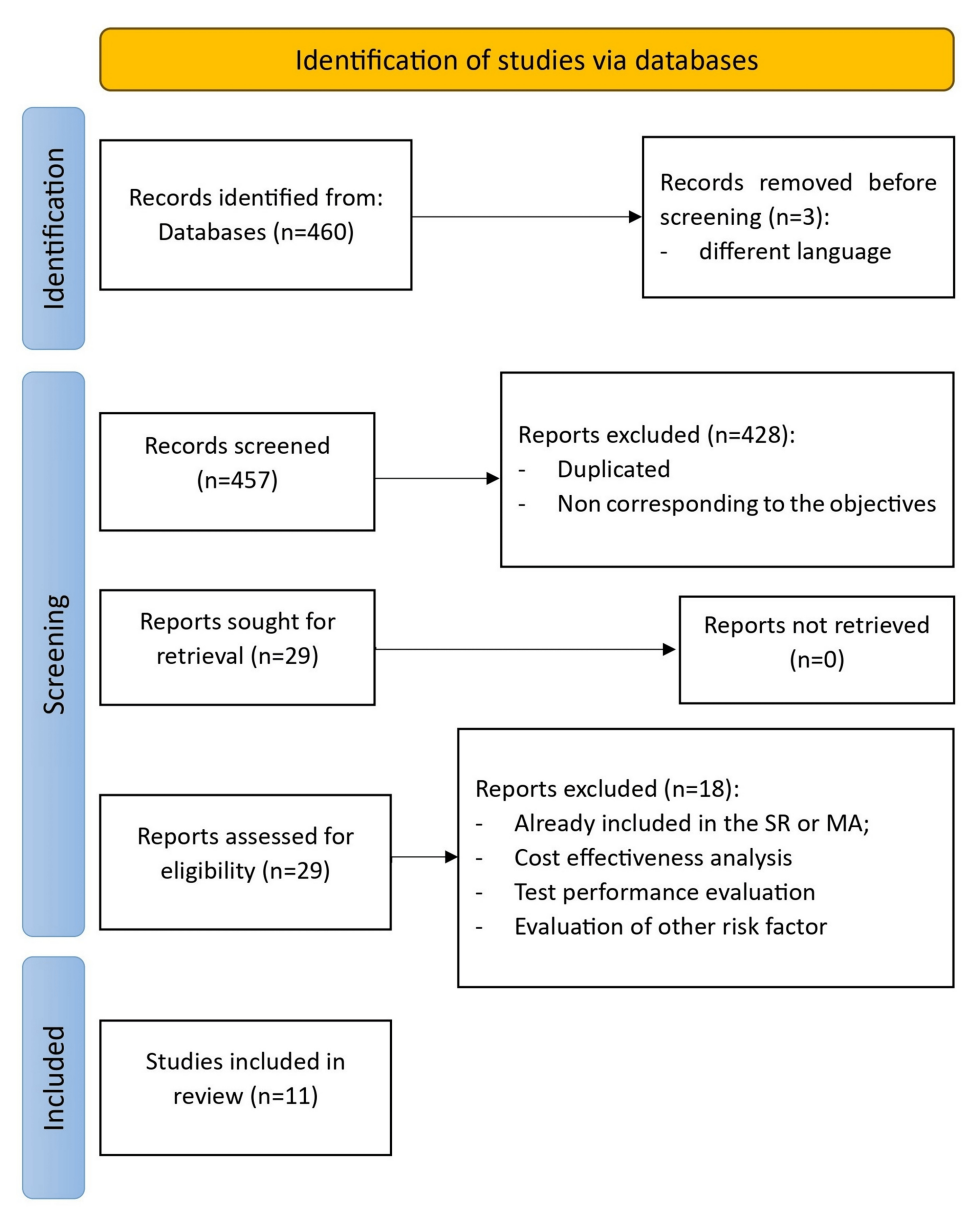
Article selection

The search identified 460 articles, of which three were excluded for not being in the languages mentioned above. By reading titles and abstracts, 428 articles were excluded for not meeting the study objectives, being duplicates, or not being RCT, NOC, MA, or SR. The selected articles were then read in full, excluding those already present in the selected SR or MA, articles that included cost-effectiveness analysis, those that evaluated other risk factors for lung cancer besides tobacco, and articles that assessed test performance (sensitivity and specificity). Of the 11 selected articles, two are NOCs, six are MAs, two are SRs, and one is RCT. The summary of the article selection process is shown in Figure 1.

Clinical Guidelines

The recommendations from the NOC are summarized in Table 1.

**Table 1:** Summary of the clinical guidelines LDCT: low-dose computed tomography

NOC	Recommendations	
Screening for lung cancer: 2023 guideline update from the American Cancer Society [11]	Annual screening for lung cancer with LDCT is recommended for asymptomatic individuals aged between 50 and 80 who currently smoke or have ever smoked and have a smoking history of ≥20 pack-years (strong recommendation; moderate quality evidence). For individuals who were once smokers, the number of years since they quit is not included as an eligibility criterion for starting or stopping lung cancer screening. Individuals with comorbid conditions that substantially limit life expectancy should not be screened. Before undergoing lung cancer screening, individuals should receive evidence-based smoking cessation counseling, be offered interventions if they currently smoke, and participate in a shared decision-making discussion with a healthcare professional about the benefits, limitations, and harms of lung cancer screening.	
Screening for Lung Cancer CHEST Guideline and Expert Panel Report 2021 [12]	*Selection: *1. For asymptomatic individuals aged between 55 and 77 who have smoked the equivalent of 30 pack-years (MU) or more and who continue to smoke or quit less than 15 years ago, it is recommended that annual screening LDCT be offered (strong recommendation, moderate-quality evidence). 2. For asymptomatic individuals who do not fulfill the smoking and/or age criteria in Recommendation #1, aged between 50 and 80, who have smoked the equivalent of 20 UMA or more and who continue to smoke or have stopped smoking in the last 15 years, it is suggested that annual screening with LDCT be offered (weak recommendation, moderate-quality evidence). 3. For asymptomatic individuals who do not meet the smoking and/or age criteria in Recommendations #1 and #2, but who are expected to have a high benefit from lung cancer screening based on the results of validated clinical risk prediction calculations and life expectancy estimates, or based on life-year gain calculations, it is suggested that annual screening with LDCT be offered (weak recommendation, moderate-quality evidence). 4. For individuals who have accumulated less than 20 pack-years of smoking, are under 50 years of age or over 80 years of age, or quit smoking more than 15 years ago, and are not expected to have a high net benefit from lung cancer screening based on clinical risk predictions or life-year gain calculators, it is recommended that screening with LDCT should not be performed (strong recommendation, moderate-quality evidence). 5. For individuals with comorbidities that substantially limit their life expectancy and negatively influence their ability to tolerate the investigation of lesions detected in screening, or to tolerate the treatment of lung cancer detected at an early stage by screening, it is recommended that screening with LDCT should not be carried out (strong recommendation, low-quality evidence).	*Implementation*: 1. It is suggested that screening programs define what constitutes a positive test on LDCT based on the size of a solid or partially solid lung nodule detected, with a threshold for a positive test being 4 mm, 5 mm, or 6 mm in diameter (weak recommendation, low-quality evidence). 2. For individuals who currently smoke and are being screened with LDCT, it is recommended that screening programs provide evidence-based smoking cessation treatment, as recommended by the United States Public Health Service (strong recommendation, low-quality evidence).

The 2023 NOC from the American Cancer Society (ACS) [11], an update of the 2013 version, recommends annual LDCT for asymptomatic individuals aged between 50 and 80 years who smoke or are former smokers with a smoking history of 20 pack-years or more. In addition, it excludes the number of years since smoking cessation as a selection criterion and excludes patients with comorbidities from screening. It also recommends that individuals entering the screening program receive a smoking cessation consultation if they are still smokers and that the decision to screen should be a shared decision with free and informed consent.

The second NOC included, from the American College of Chest Physicians (CHEST) [12], presents a series of recommendations, highlighting those with the best evidence, dividing them into two groups: one for selecting the target population and another for implementing screening. In the first group, a strong recommendation with moderate-quality evidence is that asymptomatic individuals aged between 55 and 77 years with a smoking history of 30 pack-years or more, who are still smoking or have quit within the last 15 years, should undergo annual screening. With a weak recommendation and moderate-quality evidence, there is also a recommendation for annual screening for individuals who do not meet the previous inclusion criteria, aged between 50 and 80 years, with a smoking history of 20 pack-years or more, and who are still smoking or have quit within the last 15 years, as well as for individuals who do not meet the previous inclusion criteria but are predicted, by risk calculators, to benefit significantly from screening. This society also recommends, with a strong recommendation and moderate-quality evidence, that individuals with a smoking history of less than 20 pack-years, aged below 50 or above 80, and who do not benefit from screening based on risk calculators, should not be screened. In the second group of recommendations, the only evidence-based suggestions were that the screening program should define what constitutes a positive test with LDCT based on the detection of a solid or semi-solid nodule with a size threshold for a positive test being 4 mm, 5 mm, or 6 mm in diameter (weak recommendation, low-quality evidence). It is also recommended that smokers who initiate the screening program should be referred for smoking cessation (strong recommendation, low-quality evidence).

Meta-Analyses

The summary of the MAs is presented in Table 2.

**Table 2:** Summary of meta-analyses RCT: randomized controlled trial, LDCT: low-dose computed tomography, RR: relative risk, OR: odds ratio, CI: confidence interval, NNS: number needed to screen

Meta-analysis	Population	Intervention	Results	Level of evidence
Mark H. Ebell et al. (2020) [13]	Eight RCTs; ages 49-74; at least 20 UMAs; smoking cessation of no more than 10 years	LDCT screening vs. chest X-ray screening or no screening	Lung cancer risk reduction RR 0.81 (95% CI, 0.74-0.89); reduction in death from any cause not significant (relative risk (RR) = 0.96 (95% CI, 0.92-1.01)); 20% overdiagnosis rate; number needed to screen (NNS) 250.	A
Cuiping Fu et al. (2014) [14]	Nine RCTs; ages 49-74; more than 15 years of smoking; cessation of no more than 15 years	LDCT screening vs. chest X-ray screening or no screening	Four studies analyzed all-cause mortality, with no statistically significant reduction (odds ratio (OR) = 1.18 (95% CI: 0.86-1.63)) with high heterogeneity; four studies analyzing lung cancer mortality showed a statistically significant reduction (OR = 0.84 (95% CI: 0.74-0.96)) with moderate heterogeneity; nine studies show a statistically significant increase in the diagnosis of lung cancer (OR = 3.38 (95% CI: 1.8-6.35)), with moderate heterogeneity.	B
Theresa Hunger et al (2021) [15]	Nine RCTs; ages 49-75; at least 20 UMAs; smoking cessation of no more than 15 years	LDCT screening vs. chest X-ray screening or no screening	Eight studies analyzed lung cancer mortality with a statistically significant reduction (RR = 0.88 (95% CI: 0.79-0.97)) with low heterogeneity; all-cause mortality with no statistically significant reduction (RR = 0.98 (95% CI: 0.95-1.02)) with low heterogeneity; overdiagnosis rate between 18.5% and 69.1%.	A
Alexandre Sadate et al. (2020) [16]	Seven RCTs; ages 49-74; at least 20 UMAs	LDCT screening vs. chest X-ray screening or no screening	Statistically significant 17% reduction in lung cancer mortality (RR 0.83 (95% CI: 0.76-0.91)); non-statistically significant reduction in all-cause mortality (RR 0.96 (95% CI: 0.92-1.00)); low heterogeneity; NNS 294.	A
Richard M. Hoffman et al. (2020) [17]	Nine RCTs; ages 49-74; at least 20 UMAs; smoking cessation of no more than 10 years	LDCT screening vs. chest X-ray screening or no screening	Statistically significant reduction in lung cancer mortality (RR 0.84 (95% CI, 0.75-0.93); reduction in all-cause mortality not statistically significant RR 0.96 (95% CI, 0.91-1.01); low heterogeneity; 33% overdiagnosis; NNS 265.	A
Xue Tang et al. (2019) [18]	Nine RCTs; ages 50-74; more than 15 UMAs; smoking cessation of no more than 15 years	LDCT screening vs. chest X-ray screening or no screening	Statistically significant reduction in lung cancer mortality (RR = 0.84 (95% CI = 0.75-0.95), P = 0.004) with moderate heterogeneity; non-statistically significant reduction in all-cause mortality (RR = 1.26 (95% CI = 0.89-1.78), P = 0.193) with high heterogeneity.	B

When analyzing the MA, some homogeneity can be seen in the studies included in the analyses. In all, the intervention was defined as screening with LDCT, and the control was defined as using radiography or no screening. In the MA by Ebell et al. [13], eight RCTs were analyzed, with a total of 90,745 patients, with smoking histories of at least 20 pack-years, including smokers and former smokers who had quit for less than 10 or 15 years, aged between 50 and 70. The cumulative incidence of lung cancer in the intervention group was higher with a relative risk (RR) of 1.21 (95% confidence interval (CI), 1.06-1.37). However, when only the five highest-quality RCTs were analyzed, the RR was 1.25 (95% CI 1.02-1.55). This MA reported a statistically significant reduction in mortality from lung cancer with an RR of 0.81 (95% CI, 0.74-0.89) and a non-statistically significant reduction in mortality from all causes (RR = 0.96; 95% CI, 0.92-1.01). The absolute risk reduction in mortality determined in this MA was 0.4% (2.12-1.72%), corresponding to a number needed to screen (NNS) of 250.

The study by Fu et al. [14] included studies with participants aged between 50 and 60, with a smoking history of over 15 years, smokers or former smokers who had quit for less than 10 years, including nine RCTs. Analyzing mortality from all causes, this MA included four studies with this variable, determining a non-statistically significant reduction in mortality with an odds ratio (OR) = 1.18 (95% CI: 0.86-1.63). Four studies also analyzed lung cancer mortality, with a statistically significant reduction in mortality OR = 0.84 (95% CI: 0.74-0.96). In addition, five studies analyzed the rate of false positives, with a significantly higher rate in the intervention group with OR = 41.77 (95% CI: 5.18-336.95).

In the study by Hunger et al. [15], nine studies were included, only one of which was described as of moderate quality due to randomization issues. The patients included in the studies were aged between 49 and 75 years, with smoking histories of at least 20 pack-years. In the analysis of mortality from lung cancer, eight studies with a total of 87,878 individuals were included, showing a 12% statistically significant reduction in mortality (RR = 0.88; 95% CI: 0.79-0.97). In the analysis of overall mortality, there was a trend toward reduction, but it was not statistically significant (RR = 0.98; 95% CI: 0.95-1.02). The rates of overdiagnosis in this MA ranged from 18.5% to 69.1%.

Sadate et al. [16] included 7 RCTs in their MA, with a total of 84,558 individuals. The reduction in mortality from lung cancer was 17%, statistically significant (RR 0.83; 95% CI: 0.76-0.91), while the reduction in mortality from all causes was not statistically significant (RR 0.96; 95% CI: 0.92-1.00). The NNS determined in this MA was 294.

Another MA by Hoffman et al. [17] included nine RCTs with a total of 96,559 patients, with an average age of 60 years and an average smoking history of 40 pack-years. A statistically significant reduction in mortality from lung cancer was calculated (RR 0.84; 95% CI, 0.75-0.93), determining an NNS of 265. Analyzing mortality from all causes, the reduction was not statistically significant (RR 0.96; 95% CI, 0.91-1.01). The rate of overdiagnosis in this MA was 33%.

Tang et al. [18] conducted an MA that included nine RCTs, with 38,357 individuals in the intervention group and 37,563 controls, both from high-risk populations for lung cancer, aged between 50 and 70 years. In the study of mortality from lung cancer, five studies were included, showing a statistically significant reduction in mortality (RR = 0.84, 95% CI = 0.75-0.95, P = 0.004). For the study of mortality from all causes, six studies were included in this MA, showing a non-statistically significant reduction in mortality (RR = 1.26, 95% CI = 0.89-1.78, P = 0.193).

Systematic Reviews 

Evaluating the SRs, we consider that of Jonas et al. [19], who conducted an SR by analyzing various outcomes, including mortality from lung cancer and mortality from all causes, which are the objectives of this evidence-based review. In assessing mortality from lung cancer, this SR describes results from three studies, one showing a mortality reduction with three rounds of annual LDCT compared to chest radiography (RR 0.85; 95% CI: 0.75-0.96), and an NNS of 323 over a follow-up period of 65 years. In an extended analysis of this follow-up period of 12.3 years, the reduction in mortality from lung cancer was similar. In another study included in this SR, assessing mortality from lung cancer, a mortality reduction of 0.75 (95% CI: 0.61-0.90) was calculated, with an NNS of 130 over a 10-year follow-up. In the analysis of mortality from all causes, only one study was included in this SR, which demonstrated a reduction in mortality (RR 0.93; 95% CI: 0.88-0.99), with the remaining studies analyzing this variable having very imprecise results.

Another SR included in this evidence-based review, by Bach et al. [20], evaluated three RCTs with results on the reduction of mortality from lung cancer and mortality from all causes. In mortality from lung cancer, one study showed an estimated relative reduction of 20% with LDCT compared to chest radiography. Two other studies did not show a statistically significant reduction. In none of the three studies was a statistically significant reduction in mortality from all causes found.

Randomized Controlled Trial

de Koning et al. [21] conducted an RCT to study whether the use of LDCT in male smokers reduces mortality. A 10-year reduction in mortality from lung cancer was calculated at 0.76 (95% CI: 0.61-0.94), with similar values at eight and nine years. An analysis was also performed in women, showing a mortality reduction of 0.67 (95% CI: 0.38-1.14) at 10 years, with values ranging from 0.41 to 0.52 in years seven to nine.

Discussion

This review included and evaluated two NOCs, six MAs, two SRs, and one RCT. The outcomes analyzed were a reduction in mortality from lung cancer, a reduction in mortality from all causes, and a rate of overdiagnosis.

Lung Cancer Mortality

Both NOCs [11,12] agree that screening should be offered to individuals with a significant smoking history and that screening should not be offered to individuals with smoking histories below 20 pack-years or with comorbidities. In addition, another recommendation from both NOCs is that individuals selected for screening should be referred for a smoking cessation consultation.

When analyzing the results of the included MAs and SRs, we observe that several original studies were repeatedly analyzed in various MAs and SRs, limiting the variability of results among MAs. Nevertheless, when evaluating the impact of lung cancer screening with LDCTs, all demonstrated a statistically significant reduction in mortality from lung cancer. In this evaluation, only two MAs showed moderate heterogeneity among the included studies [14,18], with the remaining showing low heterogeneity. These results validate the evidence that lung cancer screening reduces mortality from this cancer, which has been the argument for recommending the implementation of such screening in various countries.

The SRs included in this review present similar results to those of the MAs. In the work by Jonas et al., the reduction in mortality from lung cancer was statistically significant in four studies but with disparate follow-up periods among the studies. By contrast, the SR by Bach et al. [20] had only one RCT with a statistically significant result. 

In addition, the RCT by de Koning et al. [21] demonstrated a statistically significant reduction in mortality from lung cancer, evaluating various follow-up periods in men. This study separately analyzed results in women, with non-statistically significant differences. This disparate result may be related to the fact that the sample of female smokers was smaller than that obtained in men.

All-Cause Mortality

Assessing mortality from all causes, despite all studies showing a reduction, none had statistically significant results. In this outcome, heterogeneity was more varied, with MAs reporting low heterogeneity [15-17], moderate heterogeneity [14], or high heterogeneity [18]. A possible explanation for this non-significant reduction is related to the comorbidities of these individuals [17,18], especially cardiovascular diseases, the leading cause of death in smoking patients [18].

Overdiagnosis

One of the main reasons for the non-implementation of organized screenings relates to the number of false positives produced by screening and the subsequent overdiagnosis. There is still no robust evidence to define the tumor size cut-off for a positive screening result [15], which will be a factor to consider in future studies. In the evaluated MAs, the rates of overdiagnosis vary significantly, with 20% in Ebell et al. [13], rates between 18.5% and 69.1% in Hunger et al. [15], and 33% in Hoffman [17]. Despite this, these rates are not very different from the overdiagnosis rate of breast cancer [13], and the fact that mortality from all causes did not show a statistically significant difference suggests that this overdiagnosis is not associated with a higher number of deaths. However, there is still a whole other set of effects from overdiagnosis that, obviously, cannot be ignored.

Limitations of the Study

This review has some limitations. First, the relative disparity in ages and smoking histories of the various studies selected in the different MAs and SRs. Also, the different follow-up times of the various studies increase the variability of the assessment, introducing another limitation to this review. In addition, some studies compared LDCT screening with chest radiography screening, rather than with no screening. However, previous studies with chest radiography have shown that it has no benefit [15], so it may be appropriate to use chest radiography screening as a control alongside no screening. Another limitation is the possible publication bias, as smaller studies or those with negative results tend to be less published and thus not included in the MA or SR. Another bias is introduced by the fact that, in this topic, trials are not double-blinded, as it is impossible to “blind” the intervention.

There are other risk factors for lung cancer; however, in this review, two studies evaluating screening in individuals exposed to asbestos were excluded. This choice had to do with the fact that the main risk factor is smoking and because the NOC themselves issued recommendations only for smokers. It would be important to increase the level of evidence for screening in individuals with other risk factors for lung cancer besides tobacco, to better select a potential population to screen.

## Conclusions

This review concludes that lung cancer screening with low-dose chest CT significantly reduces mortality from the disease, with a grade of evidence A. As this is an important cause of death worldwide, with a relevant economic and social burden, the implementation of organized national screening programs could have a significantly positive impact on the care provided to these patients.

However, there are still some doubts, which, for the proper structuring and implementation of robust and effective screening, should be further studied, such as the exact ages of the target population to be selected, the definition of the ideal follow-up time, or the protocol for managing false positives.
